# Confident Predictability: Identifying reliable gene expression patterns for individualized tumor classification using a local minimax kernel algorithm

**DOI:** 10.1186/1755-8794-4-10

**Published:** 2011-01-24

**Authors:** Lee K Jones, Fei Zou, Alexander Kheifets, Konstantin Rybnikov, Damon Berry, Aik Choon Tan

**Affiliations:** 1 Department of Mathematical Sciences, University of Massachusetts, Lowell, MA, USA; 2 Division of Medical Oncology, Department of Medicine, University of Colorado Denver School of Medicine, Anschutz Medical Campus, Aurora, CO, USA

## Abstract

**Background:**

Molecular classification of tumors can be achieved by global gene expression profiling. Most machine learning classification algorithms furnish global error rates for the entire population. A few algorithms provide an estimate of probability of malignancy for each queried patient but the degree of accuracy of these estimates is unknown. On the other hand *local minimax learning *provides such probability estimates with best finite sample bounds on expected mean squared error on an individual basis for each queried patient. This allows a significant percentage of the patients to be identified as *confidently predictable*, a condition that ensures that the machine learning algorithm possesses an error rate below the tolerable level when applied to the confidently predictable patients.

**Results:**

We devise a new learning method that implements: (i) feature selection using the k-TSP algorithm and (ii) classifier construction by local minimax kernel learning. We test our method on three publicly available gene expression datasets and achieve significantly lower error rate for a substantial identifiable subset of patients. Our final classifiers are simple to interpret and they can make prediction on an individual basis with an individualized confidence level.

**Conclusions:**

Patients that were predicted confidently by the classifiers as cancer can receive immediate and appropriate treatment whilst patients that were predicted confidently as healthy will be spared from unnecessary treatment. We believe that our method can be a useful tool to translate the gene expression signatures into clinical practice for personalized medicine.

## Background

As developing gene expression signature from microarray data becomes a routine strategy to predict clinical outcome or to classify molecular tumor subtypes, computational methods that are capable of extracting *accurate *and *simple *decision rules from such microarray data are of great interest in *personalized *medicine [1-5]. Various gene expression signatures and classifiers have been identified recently; however, only a handful of these signatures are translated from bench research to clinical trials. Several factors contribute to this problem from the bioinformatics perspective: First, the lack of interpretability in the resulting classification rules (gene expression signatures), generated from standard machine learning approaches, leaves a mystery for clinicians. Second, the number of genes involved in the classifiers ranges from tens to hundreds; interpreting, validating, implementing and translating these classification rules to the clinician represent a daunting task. Third, in order to achieve personalized prediction due to a patient's tumor heterogeneity, the classification rules have to be able to provide *a predicted probability of malignancy *with a *level of confidence *where both quantities vary with the *individual patient*. This prediction capability is not available in current machine learning approaches [6,7]. Finally, other technical and biological issues were described in [8] that hindered the translation of gene signatures in clinical practice.

Motivated by these computational challenges, we devise a novel statistical framework for personalized prediction with accurate and simple decision rules from microarray gene expression data [9]; where for each patient we are able to give an individualized analysis. For each patient we generate a predictor vector whose k components consist of the differences of signal intensities from tissue samples from k gene pairs. For two class pattern recognition problems (with patient labels 0 for normal and 1 for cancer), we use local minimax learning to obtain both an estimate of the probability that a patient with a given predictor vector belongs to class 1 (cancer) and a confidence interval for the true probability. The length of the confidence interval varies (as does the probability estimate) with the given query so that the action taken for a given query may depend on both degree of confidence (inverse of length of confidence interval) and degree of predictability (nearness of probability estimate to 0 or 1). Indeed, in practice, classification algorithms are often only one step in a larger sequential decision procedure. Final decisions may only be reached if the classification process yields high degrees of both confidence and predictability. We define confident predictability for a patient in terms of the above two degrees and examine the machine learning algorithm performance for the confidently predictable patients. Otherwise, for non-confidently predictable patients, further measurements and testing may be desired.

A local minimax learning algorithm may be applied directly to (i) raw predictor data or to (ii) predictor data of reduced dimensionality after feature selection via virtually any favorite machine learning algorithm. However, although it is believed that local minimax learning will compete favorably with many machine learning algorithms, for case (i) difficult optimization problems need to be solved (hopefully they will be solved as a result of future computational research) to implement the techniques of optimal fusion and optimal local kernel shape determination derived in [10]. Also it is almost always the case that understanding the science behind the statistical problem leads naturally to appropriate feature selection. Hence we report here on implementations of type (ii) only.

Our framework consists of two major steps: 1) feature selection and 2) prediction and error estimation. We used the *k*-*T*op *S*coring *P*airs algorithm (*k-TSP*) [9] for feature selection yielding a k dimensional feature space with coordinates given by the gene pair intensity differences described previously. The prediction and error estimation are based on the theory of Local Minimax learning [10]. We applied the local minimax prediction algorithms based on the Improved Tikhonov Estimator for kernel machine learning to estimate a given patient's probability of having cancer. The idea of local learning is to individualize the prediction process: the determination of the probability of cancer for a patient is "tailored" to the neighborhood of the patient's predictor vector. For comparison a nearest neighbor algorithm with a (only asymptotically valid) confidence interval was similarly applied.

## Methods

### Feature selection method

We used *k*-*T*op *S*coring *P*airs (*k-TSP*) [9] algorithm as feature selection method in this study. Suppose we have *N *patients with known diagnoses (0 for normal or 1 for cancer) and for each patient we are given a vector g of real numbers consisting of the values of signal intensities of tissue samples from G *genes*. For any pair of genes (*i*, *j*) we can determine the relative frequency that g*_i _*< g*_j _*among the normal patients and also compute this relative frequency for the cancer patients. We determine a gene pair (*i*, *j*) for which the absolute difference of the two relative frequencies is greatest. Using this absolute difference, we can order all the gene pairs from the greatest to the lowest. In case of ties, we replace the signal intensities of each gene by their ranks among the *N *patients and compute the average within class rank difference for gene pairs and break the tie by maximizing the absolute class difference (See [9] for more efficient algorithms.). In this study, we fixed *k *= 10 to select the top 10 disjoint gene pairs as the learning features for the local minimax algorithm.

We note that in the feature selection the true classes of the patients are used. This will affect the local minimax probability estimate as discussed below but the width of the confidence interval will remain valid.

### Prediction and error estimation

Suppose we have *N *patients with known diagnoses and for each patient we are given a vector of *k *real numbers consisting of the values of *k *selected features. The *i*-th number represents the difference of signal intensities for the *i*-th top scoring pair. Typically *N *is in the range of a few dozen to a few hundred and we set *k *= 10 in this study. We represent the *j*-th patient vector by *x_j_*. Let *Y_j _*be 1 for those who have cancer and 0 for those who do not. A new patient is now considered and we are given his feature values in the vector *x*_0_. Let *f *(*x*) Prob {cancer | feature vector x}. We will assume that *f *(*x*) is a constant plus a finite sum of kernel functions centered at arbitrary points in a given sphere V about *x*_0 _in *k *dimensional space. (Actually this representation only needs to equal the above probabilities at *x_j _*for *j *= 0,1,..., *N *) We represent the kernel function as *K *(*z*, *x*)where *z *is the center and *x *varies over the sphere V. We used the kernel $K\left(z,x\right)=e^{-\left(\frac{0.5{\left(x-z\right)}^{t}\left(x-z\right)}{\sigma^{2}}\right)}$ although our analysis remains valid for many kernels. Here σ is a fraction of the distance of the query *x*_0 _to the furthest *x_j_*. We took that fraction as 0.5 and 0.7 in the experiments reported here. Other fixed fractions could also be used. Or one could use a fixed fraction of the mean distance from the query to a patient in the training set, or a fixed fraction of the distance to the *j*-th closest patient, etc. Cross validation for determining is not recommended as it will give a global average for which would detract from accurate local analysis.

Since taking any linear combination of kernels above will be equivalent to assuming the set of such *f *'s is dense in the set of all continuous functions, we restricted the linear combinations to be possibly probability functions (meaning 0 <*f *(*x*) < 1) and to have a degree of smoothness directly proportional to σ. In particular *f*(x) = *α *+(1- *α*)*f_1_*(x) - *αf_2_*(x) (for *α *fixed, 0 <*α *< 1) where each *f_l _*(*l *= 1,2) is a positive finite sum of kernels centered at arbitrary points {*z_i_*} ⊂*V *where each 0 <*f_l _*(x) < 1 in V. (Denote the class of such *f *'s by *PK*_α _(*V *). We took *α *= 0.5 in our experiments although we present the results for any *α*). Actually we only need to assume that there is an *f *in *PK_α _*(*V_i_*) that takes the same values as the true probabilities at the data points and query in the sphere V*_i _*below and where {*z_i_*} ⊂*V_i_*. We obtain an estimate of *f *(*x*_0_) of the form $F\left(w\right)=w*+\sum_{j=1}^{N}w_{j}Y_{j}$, together with a bound on mean squared error (MSE) and a one sided 90% confidence interval. These three quantities are furnished by the improved Tikhonov estimator (1),(2) and (3) below: (which is derived in full in [10] (and outlined in the appendix) by the method of local minimax learning).

First assume the patient training vectors are ordered by their distance to the query *x*_0_. *V_i _*is the ball centered at *x*_0 _of radius equal to the distance from *x*_0 _to the *i *-th closest training vector where *i*∈{1,2,...,*N*} is optimally chosen to minimize the bound (1). (i.e. an optimal neighborhood is chosen to which the contextual Tikhonov estimator of [10] is applied).

(1) $${£}_{i}=\frac{1}{\left[{\left(\sigma *+M_{V_{i}}^{2}K_{i}^{*}\right)}_{00}^{-1}\right]}=MSE\;bound,$$

(2) $$\left(-1,w_{1},w_{2},\ldots ,w_{i}\right)=-\frac{1st\;\;row\;\;of\;\;{\left(\sigma *+M_{V_{i}}^{2}K_{i}^{*}\right)}^{-1}}{{£}_{i}}\;\;\text{and}\;w_{i+1},w_{i+2},\ldots ,w_{N}=0$$

where $M=M_{V_{i}}={\left(\alpha^{2}+{\left(1-\alpha \right)}^{2}\right)}^{\frac{1}{2}}{\left(\underset{x\in V_{i}}{\max}\underset{y\in V_{i}}{\min}K\left(y,x\right)\right)}^{\frac{1}{2}}$, w* = -α (-1 *+ w*_1 _+ ... + *w_i_*) and (denoting by *z_β _*the (1 - *β*) -th normal quantile) (1 - *β*) confidence intervals

(3) $$\left(F\left(w\right)-{£}_{i}^{\frac{1}{2}}{\left(1+z_{\beta }^{2}\right)}^{\frac{1}{2}},+\infty \right)\;\text{and}\;\left(-\infty ,F\left(w\right)+{£}_{i}^{\frac{1}{2}}{\left(1+z_{\beta }^{2}\right)}^{\frac{1}{2}}\right)$$

where the *i *+ 1 dimensional diagonal matrix σ* = *diag*{0,0.25,0.25,...,0.25}, $K_{i}^{*}=K\left(x_{j},x_{m}\right)$, *j*,*m = *0,1,2,...,*i *. (In [10] it is proposed to further optimize by varying the kernel function appropriately over those with trace of the kernel covariance equal k/σ^2^).

In fact our estimator is identical to that furnished by first least squares fitting the data in the above *V_i _*to a function *g(x) *with a penalty term (0.5*/MV_i _*)^2 ^*[g,g] *^2^, where *[ , ] *is the reproducing kernel inner product (see appendix), and then using *g(x_o_) *as the estimate. This penalized least squares procedure is the well known Tikhonov regularization. For a proof of the identity of our estimator and a Tikhonov regularization see Theorem VI in [10].

A complete outline of Reproducing Kernel Hilbert space (RKHS) and the derivation from 3 stated local minimax results (to which the reader may give his/her own proofs) is given in the appendix. References are given to the full proofs of these three results in [10].

Now the preceding results are valid provided the feature selection process does not depend on the patient classes *Y_j _*but only on the patient raw predictor vectors g. However if we could find *N *new patients with exactly the same *N x_j_'*s as the training set then the results using the *Y_i _'*s for these new patients would be valid for the queried patient. Of course we can not find such patients easily but the MSE bounds (and hence the width of confidence intervals) depend only on the *x_j _*. Hence with top scoring pairs as a feature selection method only the probability estimators *F *(*w*) depend on the new *Y_j_*. We can approximate such *F *(*w*) by adjusting the estimator by an (expected) resampling shift and then using the invariant MSE bound to form the approximate confidence interval: First suppose the true overall total error rate with the given feature space is 0. Then the new patients (even though we can not find them in practice) will have the same *Y_j _*and the results are valid for the original training set. For a true overall total error rate of e we can generate an approximate new "adjusted" *F *(*w*) by replacing the original *Y_j _*in the formula for *F *(*w*) by the value e if *Y_j _*= 0 or 1-e if *Y_j _*= 1. Now the true overall total error rate is likely to be quite small as machine learning suffers from the curse of dimensionality. So we report results here with adjustments (e) of e = 0.00 and e = 0.05.

## Results

### Microarray gene expression datasets

To demonstrate the utility of the local minimax algorithm, we tested it on three publicly available microarray gene expression data sets. *Leukemia*. This data is described in [11] for separating ALL from AML using microarray gene expression profiles. In total, there are 47 ALL and 25 AML samples and 7,129 gene features. *Prostate Cancer*. This data set is described in [12]. The gene expression profiles contain 38 tumors and 50 normal prostate samples and 12,625 gene features. *Global cancer map (GCM)*. This gene expression data set represents a collection of 280 various tumors (190) and normal (90) samples. The classification task is to distinguish tumors from normal based on 16,063 gene features [13].

### Leave-one-out cross-validation

For all the experiments, we employed leave-one-out cross-validation to assess the classification performance. In brief, for each data set of size N, we left out one sample, and performed feature extraction and classifier constructed based on the N-1 member training set. The classifier constructed from the N-1 member training set is used to predict the left out sample. This procedure is repeated N times.

### Predictability threshold and accuracy

When applying one of our classification algorithms to a given patient's predictor vector (gene expression profile), we not only obtain an estimate F of the probability that a patient has cancer (whence we classify the patient into class 0 for normal if F is less than 0.5, or else we classify the patient as class 1 for cancer), but we also obtain a 90% one-sided confidence interval for that patient's predicted probability. Since we are estimating a probability -∞ and +∞ may be replaced in the confidence interval expressions (3) by 0 and 1 respectively.

One use of such an algorithm is to divide patients into a group of those for whom we may confidently predict and those for whom lack of sufficient confidence or predictability may warrant further (possibly invasive) testing. This dichotomy is achieved using what we call a predictability threshold p as follows: we fix a probability value, call it p; for example p = 0.35. We then designate as confidently predictable at level p, all patients whose confidence interval associated with their prediction lies entirely inside of the interval [0, p] or lies entirely inside the interval [1-p, 1]. All other patients are considered to be non-confidently predictable. It follows that if a patient is confidently predictable (CP) with threshold p then either [0, p] or [1-p, 1] contains the true probability with 90% or more confidence.

Table 1 summarizes our results using *10-Top Scoring Pairs *for feature selection on the three microarray gene expression data sets. Predictions for each patient were made based on their raw predictor vector using the raw predictor and response data for the remaining patients ("leave-one-out" method). That is, the feature space was determined using the raw data for the remaining patients.

**Table 1:** Kernel method, sigma = 0.5 and 0.7, threshold, p = 0.35, adjustment, e = 0.00 and 0.05

Data set	Sigma	Adjustment (e)	CP (%)	Error in CP (%)	Total Error (%)
**Leukemia**	0.5	0	36 (50.0%)	0 (0%)	3 (4.17%)
		
		0.05	25 (34.7%)	0 (0%)	3 (4.17%)
	
	0.7	0	43 (59.7%)	0 (0%)	3 (4.17%)
		
		0.05	36 (50.0%)	0 (0%)	3 (4.17%)

**Prostate**	0.5	0	32 (36.4%)	4 (12.5%)	21 (23.9%)
		
		0.05	30 (34.1%)	3 (10.0%)	21 (23.9%)
	
	0.7	0	34 (38.6%)	4 (11.8%)	22 (25.0%)
		
		0.05	25 (28.4%)	2 (8.00%)	22 (25.0%)

**GCM**	0.5	0	154 (55.0%)	6 (3.90%)	39 (13.9%)
		
		0.05	134 (47.9%)	3 (2.24%)	39 (13.9%)
	
	0.7	0	160 (57.1%)	6 (3.75%)	42 (15.0%)
		
		0.05	134 (47.9%)	5 (3.73%)	42 (15.0%)

Sigma (σ) denotes the bandwidth of the Gaussian kernel in units of distance in predictor feature space from the queried patient's predictor to the furthest predictor among the rest of the patients. We report two sigma values (0.5 and 0.7) using the threshold p = 0.35 on these data sets in Table 1. In Table 1, we reported the results of e = 0.00 and 0.05 as adjustments to *F *(*w*).

From Table 1, we observed that, with 10 pair differences as features, more than 50% of the AML/ALL patients may be identified as confidently predictable and that their error is reduced from 4% to 0%. Similarly 55% of the GCM patients may be separated and their error is reduced from 14% to less than 4%. For the prostate data set about one-third of the patients get their error rate cut by 70%.

### Varying sigma (σ)

**Figure 1:**
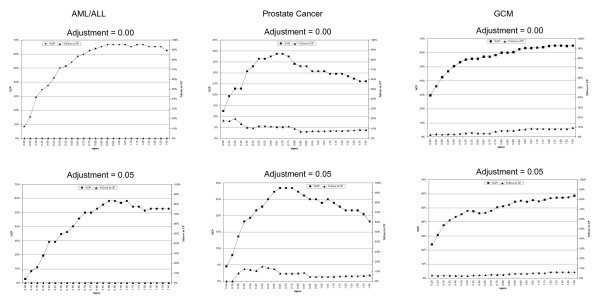
**The relationships between sigma, percentage of confidence predictable (CP) patients and the percentage of error in CP patients in the three microarray data sets**.

In Figure 1 we plot %CP and %error in CP as a function of sigma from 0.2 to 1.4. In general confidence widths may be narrower for larger sigma but the true probability function must be assumed to be very smooth.

### Confident predictability with 3-nearest neighbor predictor

We only demonstrate improved classification for the kernel classifier using squared error loss. Finite sample local accuracy bounds are not available for other machine learning algorithms. However, for nearest neighbor algorithms, we may use asymptotic properties to define confident predictability and compare with our finite sample methods. The 3-nearest neighbor algorithm represents a simple local learning method whose predictions represent a baseline predictive power of asymptotic local learning approaches. The gain of our proposed methods can be assessed by comparing to the 3-nearest neighbor predictor. For this purpose, we implemented a 3-nearest neighbor predictor in this study. Employing the same leave-one-out cross validation procedure as previously described, we compared the 3-nearest neighbor predictor against the kernel predictors with sigma 0.5 and 0.7 on the three microarray data sets. Assuming the true probability of cancer for any patient is either at most 0.30 or at least 0.70, confident predictability for p = 0.35 (as sample size approaches infinity) can be defined as "all 3-nearest neighbors belong to the same class". Table 2 shows the results of the comparisons of CP (%), error in CP (%) and total error(%).

**Table 2:** Leave-one-out comparisons of the local minimax learning with 3-nearest neighbor predictor and the kernel predictors (sigma = 0.5 and 0.7, p = 0.35).

	3-nearest neighbor predictor			Kernel predictor with sigma = 0.5, e = 0			Kernel predictor with sigma = 0.7, e = 0		
	
Data Set	CP (%)	Error in CP. (%)	Total Error (%)	CP (%)	Error in CP (%)	Total Error (%)	CP (%)	Error in CP (%)	Total Error (%)
**Leukemia**	71	3	3	36		3	43		3
	(98.6%)	(4.23%)	(4.17%)	(50.0%)	0 (0%)	(4.17%)	(59.7%)	0 (0%)	(4.17%)

**Prostate**	54	11	21	32	4	21	34	4	22
	(61.4%)	(20.4%)	(23.9%)	(36.4%)	(12.5%)	(23.9%)	(38.6%)	(11.8%)	(25%)

**GCM**	204	15	38	154	6	39	160	6	42
	(72.9%)	(7.35%)	(13.6%)	(55.0%)	(3.9%)	(13.9%)	(57.1%)	(3.75%)	(15%)

As indicated in Table 2, the local minimax kernel methods had a greatly reduced % error in CP while the 3-nearest neighbor predictor had little or no reduction in two cases and, in the remaining case, had a CP (%) error nearly double that for the kernel method. This is expected since the 3-nearest neighbor confidence depends on virtually infinite sample size while local minimax learning bounds are optimal for finite samples.

### Application to individual patients

One of the novel contributions of the current method is its ability to provide both predictive power and confidence level for individual patients. We selected four patients from the GCM data set to illustrate these unique features of personalized prediction. For the patients in the GCM data set, the kernel method for 10-TSP with sigma = 0.5 was used. In this data set, we applied confident predictability (CP) with the assumption p = 0.35. We took the adjustment parameter e = 0.

### Patient 12

Based on the gene expression profile of this patient's tissues, they are correctly predicted to be cancerous with an estimated probability of 0.962. The square root of the mean square error bound (RMSE) is 0.081 (90% CI, 0.831 to 1.0). This patient is in the confidently predictable group. Given a prediction with this accuracy, a physician can initiate the treatment for this patient without further invasive diagnostic tests which might cause the disease to spread more rapidly. Giving the right treatment at the right time may stabilize the tumor cells.

### Patient 209

From the gene expression profile, the kernel algorithm (correctly) predicted this normal patient's tissue as cancerous with an estimated probability of 0. The RMSE was 0.209 and the confidence interval was [0, 0.226]. This is considered a confident prediction. In the clinical setting, this patient can be assured by the physician that no further diagnostic tests are required in the near future.

### Patient 244

The predicted probability for cancer was 0.579 with RMSE of 0.072. The 90% CI was [0.462, 1.0]. Although the RMSE was quite small the true probability of cancer was too close to 0.5 to make a firm decision. The patient may be advised by the physician to undertake further different noninvasive diagnostic tests. This non-confidently predictable patient did not have cancer but would have been classified as cancer if the decision were based on the probability estimate alone.

### Patient 253

The predicted probability for cancer was 0.837 with RMSE of 0.159. Although the probability estimate was quite high the RMSE was more than double that of patient 244 and the confidence interval was [0.579, 1.0]. This patient may be advised by the physician to undertake further different noninvasive diagnostic tests. This non-confidently predictable patient did not have cancer but would have been certainly been diagnosed as having cancer if the decision were based on the probability estimate alone.

**Figure 2:**
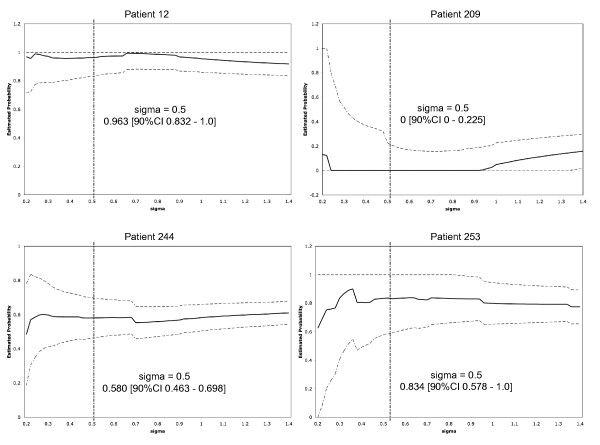
**Estimated predicted probability and 90% confidence intervals (90% CI) for the four patients in GCM data set**.

We also plotted the probability estimate and upper and lower confidence curves as a function of sigma for the four patients in Figure 2. In general the user may want to set a lower limit on sigma - say the fraction given by the distance to the (N/5)-th closest predictor vector divided by the distance to the furthest. Except for patient 209 (whose fraction was 0.42) these lower limits were less than 0.2.

## Discussion

We have developed a local minimax kernel learning algorithm that is capable of making individualized prediction in several microarray cancer gene expression data sets. This method incorporates two learning algorithms: the unique features of the *k-TSP *algorithm by retaining its simple and accurate decision rules, and adding estimated probability and confidence interval for individualized prediction from a local minimax kernel learning algorithm. Moreover, our predictions can be made on an individual basis with local estimated probability and confidence level, which are currently not available in the predictions of other learning methods.

Cancer is heterogeneous in nature, where every patient's tumor harbors different genetic alterations, even though they have the same cancer type [14]. Understanding these genetic variations between individuals could have a profound impact in combating this disease; as one of the major clinical challenges is to identify a subset of patients who could benefit from certain type of chemotherapies, both in metastatic and adjuvant settings [1,2].

Machine learning or statistical learning approaches have been widely used to classifying and stratifying cancer patient data based on gene expression data [5-7]. In general, current machine learning classifiers only report global error rates over a large population [5-7]. Such rates are useful for epidemiologists and hospital administrators trying to predict future trends, loads, and disease rates. However, in a clinical setting, a global error rate is of little comfort to the patient and his or her doctor. This is where our methods have a distinct advantage. Since we are able to label our predictions as either being confidently predicted or non-confidently predicted, these methods have the potential to be more powerful in the context of sequential analysis than the current standard methods.

Physicians requiring a greater level of confidence may decide to classify using a smaller predictability threshold. Similarly, doctors seeking to be able to confidently predict for a larger number of patients may choose to use a slightly higher threshold. These values may be investigated and adjusted over time so as to be of the most appropriate usefulness given whatever analytical context the doctor is working in at that time. Thus, by providing the physician with not only the prediction, but also with a level of confidence, and with error bounds associated with that prediction, we are able to empower the physician to make use of a larger number of more finely-grained protocols that he or she may follow as regards the care of his or her patient. This allows the physician many more options when designing how they want to sequentialize their various treatment options for various types of patients given various observations.

It must be emphasized that we only demonstrate improved classification for the kernel classifier using squared error loss. The analogous finite sample local accuracy bounds are not available for other methods (with the exception of linear regression [10]). So a very important question is whether similar localization results which identify confident prediction are valid for other algorithms. To this end we formulate important new open mathematical questions whose solutions would characterize confident predictability for a local version of kernel learning with hinge loss (Support Vector Machine- SVM), for single hidden layer neural network local learning with squared error loss, and for logistic regression local learning with squared error loss. Solution to these questions will provide both the opportunity to compare various algorithms for individualized performance as well as lead to possible fusion of several algorithms into an algorithm with superior global performance. For instance a classifier which applies both a neural net and a local kernel algorithm to a queried patient, making a decision based on the more confidently predictable output, could have a global error rate significantly lower than either classifier.

## Open Problems

In this study we have only applied finite sample local minimax bounds for Tikhonov kernel learning (i.e. using squared error loss) and obtained improved accuracy. An important open problem is to obtain local accuracy bounds for the support vector machine (which uses hinge loss and fits linear combinations of the kernel with prescribed bandwidth σ while penalizing by adding a constant times square of the reproducing kernel norm of the linear combination) and examine the improvement via confident predictability for that machine. One approach to this problem is to optimally map the SVM discriminant function *g(x) *to the interval 0[1] by *s(g(x)) *yielding a probability of cancer estimate and then obtain results on the mean squared error of that estimate at *x*_o_. Also open is the local minimax bound with squared error loss for logistic regression or more generally for a probability of cancer expression which is a ridge function. Here consider the same affine estimators of the form $F\left(w\right)=w^{*}+\sum_{j=1}^{N}w_{j}Y_{j}$, but assume *f(x) *is a sigmoidal function *exp*{*a*_o _+ *a***·***x*}/(1 + *exp*{*a*_o _+ *a***·***x*}) with a prescribed bound on the length of *a*, and then get a bound on the mean squared error at *x*_o_. We might also do this when *f *is of the form *h*(*a*_o _+ *a***·***x) *,where *a *is a unit vector, for some class of univariate *h*'s. Finally a local accuracy bound is desired for the single hidden layer neural network and its potential for extending confident predictability to neural network classifiers. Here consider again affine estimators but assume *f(x) *is a convex combination of *n *sigmoidal functions, where the i'th is of the form *exp*{*a*_i,o _+ *a*_i_**·***x*}/(1 + *exp*{ *a*_i,o _+ *a*_i_**·***x *}) with a prescribed bound on the lengths of the *a*_i_, and then get a bound on the mean squared error at *x*_o_.

## Conclusions

In summary, we have devised a new learning method that implements: (i) feature selection using the k-TSP algorithm and (ii) classifier construction by local minimax kernel learning. We tested our method on three publicly available gene expression datasets and achieved significantly lower error rate for a substantial identifiable subset of patients. Our final classifiers are simple to interpret and they can make prediction on an individual basis with an individualized confidence level. We believe that our method can be a useful tool to translate the gene expression signatures into clinical practice for personalized medicine.

## Appendix

We will outline the derivation of the bounds and algorithms for the case *i *= *N *(all *N *training vectors are used). The results are clearly also valid for *i *= 1,2,..., *N *. We suppress the subscript *N *and just use the notation *V*, *K*,σ*, £ etc.

Consider the pre-Hilbert space of models *f *(*x*;*a*) = Σ*a*_*x*_·K(*x*', *x*) where the sums are initially over finitely many *x *;where *K *(*u*,*v*), is a piecewise continuous, bounded symmetric, non-negative kernel function on *V *× *V *, positive at diagonal points (*u u*); and where the matrix *K *(*z_i_*, *z_j_*) is positive semi-definite for any finite {*z_i_*} ⊆ *V *(positive definite for distinct *z_i _*). Define an inner product [*f *(*x*;*a*), *f *(*x*;*b*)] = ΣΣ*a*_*x'*_*b*_*x''*_*K*(*x*',*x*''). Now extend this to form a real Hilbert space by completion. For any g in the constructed Hilbert space, g can be identified with the point wise limit of a sequence of models in the pre-Hilbert space which converges to g in the constructed Hilbert space. It can easily be shown that [*g*,*K*(*u*,-)] = *g *(*u*), where *g *(*u*) is the value of the associated point wise limit at *u *. Hence the space is called a reproducing kernel Hilbert space (RKHS). Assume, throughout that || || equals RKHS norm and that there are *N *predictors {*x_i_*}⊆ *V *.

Let *M *= {g: the RKHS norm of g is less than or equal to M}. By translation we may assume that the query vector *x*_0 _= 0. The following two theorems are proven in [10], for the more general case of *f *(*x*) being within *ε*(*x*) in V (where *ε *(0) = 0) of some member of the family *M*.

### Theorem I (Minimax Query-based Vector Machine)

Let *f *(*x*) be any function (not just a "probability of class 1 given *x*" function) in *M, Y_j _= f *(*x_j_*) *+ N_j _*, *j *= 1,2,..., *N *and noise covariance matrix **N **the bounded (in the semi-definite order - i.e. **σ **- **N **is positive semi-definite) by a positive definite **σ **(in this paper **σ **= 0.25 **I**). Consider the matrix K* = ((*K *(*x_i_*,*x_j_*))):*i *, *j *= 0,1,2,..., *N *.(V is centered at the query point *x*_0 _which we are taking as 0 but the results obtained are the same for any query point *x*_0 _by changing *x*_0 _to the origin and subtracting *x*_0 _from each predictor *x_j_*). Set *w*_0 _= -1 ( *w *has now *N *+ 1 components), σ* equal the *N *+ 1 by *N *+ 1 matrix formed by adding a 0-th row and 0-th column of zeros to the noise covariance matrix upper bound **σ**, and the *N *+ 1 dimensional vector *u *= (1,0,0,...,0)*^t^*. Let $£=\frac{1}{u^{t}{\left(\sigma^{*}+M^{2}K^{*}\right)}^{-1}u}$.

Then the mean squared error of *F*(*w*), where w* = 0 (Note *F *(*w*) does not involve *w*_0_), is bounded by £ if $w=-\frac{{\left(\sigma^{*}+M^{2}K^{*}\right)}^{-1}u}{u^{t}{\left(\sigma^{*}+M^{2}K^{*}\right)}^{-1}u}$ and this is the best possible bound on mean squared error if we allow any noise covariance **N **bounded in semi definite order by **σ**.

Proof: see theorem VI in [10].

### Theorem II (Vector Machine with Context)

Assume hypotheses and notation of Theorem I except *f *(*x*) takes values in 0[1] and is in *PK_α _*(*V *). Then the estimator *F *(*w*), which equals *F *(*w*) of Theorem I except that w* = -*α *(*w*_0 _+ *w*_1 _+ ... + *w_N_*), has mean squared error bounded by £ of Theorem I

when

$$w=-\frac{{\left(\sigma^{*}+M^{2}K^{*}\right)}^{-1}u}{u^{t}{\left(\sigma^{*}+M^{2}K^{*}\right)}^{-1}u}$$

where we have

$$M=M_{V}={\left(\alpha^{2}+{\left(1-\alpha \right)}^{2}\right)}^{\frac{1}{2}}{\left(\underset{x\in V}{\max}\underset{y\in V}{\min}K\left(y,x\right)\right)}^{\frac{1}{2}}\geq \sup_{PK_{\alpha }\left(V\right)}\left(\left\|g\left(x\right)-\alpha \right\|\right).$$

For such *M *, we call *F *(*w*) the contextual Tikhonov estimator. In fact, for any *M *greater than or equal the right hand side of the above inequality, the same result holds. A good choice for *α *is 0.5 (which is used in our experiments) since it minimizes *M_V _*as a function of *α*.

Proof: see theorem VII of [10].

### Confidence analysis

As *F *(*w*) is near normal in many situations according to the Lindeberg-Feller theorem, we can give approximate confidence intervals for *f *(0). Let *F *(*w*) be normal with mean *μ*, standard deviation σ and root mean squard error *ρ*. For *β *< 0.5 denote by *z_β _*the (1 - *β*)'th normal quantile. Then Pr {*F *(*w*) - *μ *≤ *σz_β_*} = 1 - *β *= Pr {*F*(*w*) - *f *(0) ≤ *σz_β _*+ *μ *- *f *(0)} $=\mathrm{Pr}\left\{F\left(w\right)-f\left(0\right)\leq {\left(\rho^{2}-x^{2}\right)}^{\frac{1}{2}}z_{\beta }+x\right\}$, with *x *= *μ *- *f *(0). (note that *ρ*^2 ^- *x*^2 ^= σ^2^).

Now maximize the right hand side inside the brackets as a function of *x *and we obtain $\mathrm{Pr}\left\{F\left(w\right)-f\left(0\right)\leq \rho {\left(1+z_{\beta }^{2}\right)}^{\frac{1}{2}}\right\}\geq 1-\beta .$.

The inequality $\mathrm{Pr}\left\{F\left(w\right)-f\left(0\right)\geq -\rho {\left(1+z_{\beta }^{2}\right)}^{\frac{1}{2}}\right\}\geq 1-\beta$ is derived in an identical fashion. If *ρ *is just an upper bound on the actual root mean squared error, the inequalities remain valid. Hence for such *ρ *we have level (1 - *β*) confidence intervals $\left(F\left(w\right)-\rho {\left(1+z_{\beta }^{2}\right)}^{\frac{1}{2}},+\infty \right),\left(-\infty ,F\left(w\right)+\rho {\left(1+z_{\beta }^{2}\right)}^{\frac{1}{2}}\right)$ and (using the inequality) $F\left(w\right)\pm \rho {\left(1+z_{\beta /2}^{2}\right)}^{\frac{1}{2}}$. In particular a 95% confidence interval is *F *± 2.22*ρ *, instead of the usual *F *± 1.96σ = *F *± 1.96*ρ *for unbiased *F *. Here we use the 90% confidence interval (*F *(*w*) - 1.621*ρ*, + ∞) or (-∞,*F *(*w*) + 1.62*ρ*).

## Authors' contributions

LKJ and ACT proposed the research, designed the study and supervised the project. LKJ, AK and KR derived the mathematical theorems and proofs. LKJ, ACT, FZ and DB implemented the algorithms, performed the analysis and interpreted the results. LKJ, ACT and FZ wrote the manuscript. All authors have read and approved the manuscript.

## Pre-publication history

The pre-publication history for this paper can be accessed here:

http://www.biomedcentral.com/1755-8794/4/10/prepub
